# Estragole and methyl-eugenol-free extract of *Artemisia dracunculus* possesses immunomodulatory effects

**Published:** 2016

**Authors:** Seyyed Meysam Abtahi Froushani, Leila Zarei, Hadi Esmaeili Gouvarchin Ghaleh, Bahman Mansori Motlagh

**Affiliations:** 1 *Department of Microbiology, Veterinary Faculty, Urmia University, Urmia, Iran*; 2 *Solid Tumor Research Center, Urmia University of Medical Sciences, Urmia, Iran*

**Keywords:** *Artemisia dracunculus (tarragon)*, *Humoral immunity*, *Cellular immunity*, *Macrophage*

## Abstract

**Objective:**

Some evidence suggests that chronic uptake of estragole and methyl-eugenol, found in the essential oil of *Artemisia dracunculus* (tarragon), may be associated with an increased risk of hepato-carcinogenicity. The present study was conducted to investigate the immumodulatory and anti-inflammatory potentials of estragole and methyl-eugenol free extract of tarragon.

**Materials and Methods:**

Aqueous, hydroalcoholic, methanol and hexane extracts of dried and milled tarragon was prepared and analyzed by GC-MS. The estragole and methyl-eugenol free extract was characterized and used for evaluation of immunity in NMRI mice after challenging with sheep red blood cells.

**Results:**

It was shown that the aqueous extract of tarragon was free from potentially harmful estragole or methyl-eugenol. Moreover, the immunomodulatory effect of the aqueous extract of tarragon (100 mg/kg for 21 consecutive days) was investigated. The extract significantly increased the level of anti-sheep red blood cells (SRBC (antibody and simultaneously decreased the level of cellular immunity in the treatment group. Moreover, tarragon caused a significant reduction in the production of pro-inflammatory IL-17 and IFN-γ in parallel with a reduction in the ratio of INF-γ to Il-10 or IL-17 to IL-10 in the splenocytes. In addition, the levels of the respiratory burst and nitric oxide production in peritoneal macrophages were significantly decreased. Additionally, the phagocytosis potential of macrophages was significantly increased in treated mice.

**Conclusion:**

These data showed that the aqueous extract of tarragon may be used as a natural source to modulate the immune system, because it can inhibit pro-inflammatory cytokines and induce anti-inflammatory macrophages.

## Introduction

*Artemisia dracunculus* (Tarragon or Tarkhun) is a perennial aromatic herb from Asteraceae family (Sayyah et al., 2004 ▶; Weinoehrl et al., 2012 ▶). It has been used in folk medicine for the treatment of cough, cold, fever, pain, amenorrhea and dysmenorrhea (Aglarova et al., 2008 ▶; Obolskiy et al., 2011 ▶). Dried aerial parts of this plant are orally prescribed in Iranian folk medicine to control epilepsy, coagulopathy and hyperlipidemia (Sayyah, et al., 2004 ▶; Shahriyary and Yazdanparast, 2007 ▶). This herb also possesses antifungal and antioxidant (Kordali et al., 2005 ▶) activity as well as anti-bacterial, hepatoprotective properties (Nageeb et al., 2013 ▶) and insecticide and radical-scavenging activities (Sayyah et al., 2004 ▶). 

Nowadays, several anti-inflammatory and immunosuppressive drugs are routinely used in order to treat inflammation-related diseases. However, long-term administration of these drugs can be associated with a high incidence of adverse side effects (Menichini et al., 2009 ▶; Saraiva et al., 2011 ▶). Therefore, medicinal plants with anti-inflammatory immunomodulatory properties and less side effects can offer a new insight in the folk medicine (Visavadiya et al., 2009 ▶). Despite popular belief, the use of medicinal plants is not always safe. For example, it has been revealed that chronic uptake of estragole and methyl-eugenol, the two compounds found in some herbs such as tarragon, was associated with an increased risk of hepato-carcinogenicity in rodents (De Vincenzi et al., 2000 ▶). Therefore, it was suggested that ingestion of products containing estragole and methyl-eugenol should be minimized (De Vincenzi et al., 2000 ▶; Weinoehrl et al., 2012 ▶).

The aim of the present work was to investigate the immumodulatory anti-inflammatory potentials of the estragole and methyl-eugenol free extract of *A. dracunculus*.

## Materials and Methods

**Chemicals**

Chemicals were provided from Sigma Chemical Co. (St. Louis, MO, USA). All the culture media were purchased from GIBCO/Life Technologies Inc. (Gaithersburg, MD, USA).

**Animals**

Male NMRI mice (5–6 weeks old, 20–25 g) were obtained from the animal center of the veterinary faculty of Urmia University, Urmia, Iran. Animals were housed under standard laboratory conditions of optimized light (12 hr: 12 hr light:dark cycle), humidity (55-60%), and temperature (22-23˚C) and received food and water *ad libitum*. Animal welfare was observed in compliance with the regulations of the Ministry of Health of I. R. Iran approved by the Medical Ethics Committee for Animal Studies.

**Plant material and Extraction**

The fresh aerial parts of *A. dracunculus* were collected from Karaj, Iran by an herbalist of the faculty of science, Urmia University (Herbarium code:512). The plant was washed, cut into small pieces, and shade-dried. Aqueous, hydroalcoholic (50% ethanol and 50% distilled water, v/v), methanol and hexane extract of the dried and milled plant was prepared using the percolation method in three steps. Finally, the extract was dried by evaporation at 40 °C. The extract was then stored without light exposure at −20°C.

**GC-MS analysis**

In order to determine the percent of estragole and methyl-eugenol present in the extract, GC-MS analysis was performed using a PerkinElmer GC Claurus 500 system and gas chromatograph interfaced to a Mass Spectrometer equipped with Elite-1 fused silica capillary column (30 m × 1µ Mdf. Composed of 100% dimethyl poly siloxane). For GC-MS detection, an electron ionization energy system with ionization energy of 70 eV was applied. Helium gas (99.999%) was used as a carrier gas at a flow rate of 1 ml/min. The injector and detector temperature were kept at 200 and 300°C, respectively. The oven temperature was programmed at 60°C (4 min), and then, raised to 300°C at 4°C/min. Mass spectrum was taken in the electron impact ionization (EI) mode with 70 eV energy with MS transfer line at 300°C. Total GC running time was 34 min. The concentration of estragole and methyl-eugenol was expressed relative to the internal standard, thymol. The limit of detection (LOD) was <1.50 ppm for methyl-eugenol and <1.05 ppm for estragole, respectively. The limit of quantification (LOQ) was 2.30 ppm for methyl-eugenol and 1.58 ppm for estragole, respectively.

**Immunological evaluation**

Mice were randomly divided into two groups of treatment and control of 10 mice. All mice were intraperitoneally immunized twice with one week interval by 1 × 10^9^ sheep red blood cells (SRBC). Mice in the treatment group received the aqueous extract of *A. dracunculus* (100 mg/kg daily) orally from the beginning of the study (onset of immunization) until the end. Animals in treatment group received placebo (PBS). Mice were bled from their hearts 7 days after the last injection and the levels of anti-SRBC antibody was estimated using the microhemagglutination test as described previously (Nelson and Mildenhall, 1967 ▶). 

Furthermore, 48 hr before bleeding time, 1 × 10^9^ SRBCs in 50 μl PBS were injected subcutaneously into the left hind foot pad of each mouse and simultaneously the same volume of PBS was administrated into the right foot pad as a negative control. 

Footpad thickness was determined before bleeding time with a dial caliper and the mean percentage increase in footpad thickness was measured according to the following formula: [(Thickness of the left footpad) _ (Thickness of the right footpad) × 100] / (Thickness of the right footpad. (

**Splenocytes isolation and cytokine production**

Spleen cells were aseptically isolated from mice at bleeding time. In brief, single-cell suspensions of splenocytes were prepared in RPMI 1640 medium supplemented with 10% fetal calf serum and red blood cells (RBCs) were removed by RBC lysis buffer. Then, cell suspensions (2 × 10^6^ cells/ml) were incubated in 24-well plates and pulsed with 50 μL phytohemagglutinin (PHA) solution (1 mg/ml). The culture supernatants were collected after 72 hr. IFN-γ, IL-17 and IL-4 productions were measured by ELISA (Bender MedSystems, Vienna, Austria) according to the manufacturer's instructions. 

**Macrophage isolation and nitric oxide assay**

Resident macrophages were isolated from the peritoneal cavity of the male NMRI mice by injection of 20 ml of ice cold PBS. The peritoneal fluid was withdrawn and centrifuged at 600 g for 10 min at 4°C. The pellets were rinsed twice in PBS and suspended in RPMI-1640 medium containing 10% heat-inactivated FCS. Cells were counted using Neuber’s chamber and the viability of the cells was evaluated by Trypan blue dye exclusion. Then, 1 ml of live cell suspension (2 × 10^6^ cell/ml) was pre-incubated in 24-well flat-bottomed plates for 40 min in a humidified incubator with 5% CO_2_ at 37 °C. This process promoted macrophage adherence to the plate. Non-adherent cells were discarded by washing the plate twice with PBS. Next, macrophages were stimulated with LPS (10 μg/ml) for 24 hr. After this period, nitric oxide production was determined using the Griess method. Cell-free supernatant (50 μl) was collected and mixed with 50 μl Griess reagent (0.1% sulfanilamide, 3% phosphoric acid and 0.1% naphthylethylenediamine) and incubated at room temperature for 10 min in the dark. After incubation, absorbance was read at 540 nm on a microplate reader (Dynatech, Denkendorf, Germany). Nitrite concentration was estimated based on a standard curve. 

**Macrophage phagocytosis**

This assay was performed as previously described (Grando et al., 2009 ▶). Macrophages (2 × 10^6^ cells/well) were cultured with Neutral Red-stained zymosan. After 30-min incubation, the supernatant was removed and Baker’s formol-calcium solution was added to stop zymosan phagocytosis. The cells were washed twice with PBS. The internalized NR was solubilized after 30-min incubation by mixing 1 ml of 10% acetic acid plus 40% ethanol. The optical density was measured at 550 nm.

**Respiratory burst potential of macrophages**

Intracellular generation of reactive oxygen species (ROS) was measured by NBT reduction as previously described (Nabi et al., 2005 ▶; Abtahi Froushani and EsmailiGourvarchinGaleh, 2014 ▶). In brief, the cells were incubated for 30 min at 37^o^C and then, an aliquot of NBT solution was added to the cells and incubated for 1 hr at 37^o^C. The unused NBT was removed through washing and the reduced dye was extracted in dioxin and quantitated at 520 nm.

**Statistical analyses**

Data was analyzed using Kruskal–Wallis test and presented as means ± SD. p-values less than 0.05 were considered statistically significant.

## Results

As shown in Table 1, the aqueous extract of tarragon did not contain any detectable amounts of estragole or methyl-eugenol. Nevertheless, other extracts contained a considerable amount of either estragole or methyl-eugenol (Table 1). Since the aqueous extract was selected and used for the following animal challenges.

**Table 1:** Concentration of estragole and methyl-eugenol in different extracts of tarragon extracts

	**aqueous **	**Hydro-** **alcoholic**	**Methanol**	**Hexane **
**Estragole**	<LOD1 ▶	1.2 ppm	1.48 ppm	1.9 ppm
**Methyleugenol**	<LOD	7.1 ppm	7.86 ppm	8.4 ppm

Aqueous, hydroalcoholic (50% ethanol and 50% distilled water, v/v), methanol and hexane extracts of the dried and milled plant were analyzed by GC-MS.

1 The limit of detection (LOD) was <1.50 ppm for methyl-eugenol and <1.05 ppm for estragole, respectively.

Footpad thickness after challenge with SRBC was used as an indicator of delayed type of hypersensitivity (DTH) reaction. Our findings indicated that mice treated with the aqueous extract of tarragon showed significantly lower DTH responses than the control group. Conversely, mean antibody titer in the treatment group was significantly higher than the mean antibody titer in control mice (Table 2).

**Table 2:** Effects of the aqueous extract of *Artemisia dracunculus* on humoral and cellular (percentage of footpad thickness) immunity

**Group**	**Antibody titer**	**Percentage of footpad thickness**
**Treatment**	239 ± 10.24	20.06 ± 4.32
**Control**	52.08 ± 4.13	55.9 ± 3.32
**p value**	< 0.001	< 0.01

Mice were intraperitoneally immunized twice with one week interval by 1×10^9^ SRBCs. Three days after the last intraperitoneal immunization, 1 × 10^9^ SRBCs in 50 μl of PBS were administered subcutaneously into the left hind foot pad of each mouse and the same volume of PBS was injected into the right foot pad as a negative control. Footpad thickness was measured and the mean percentage increase in footpad thickness was calculated. Mice were bled 5 days after the last intraperitoneal injection and the levels of anti-SRBC antibody were measured by microhemagglutination test. The values were presented as mean ± SD

**Figure 1:**
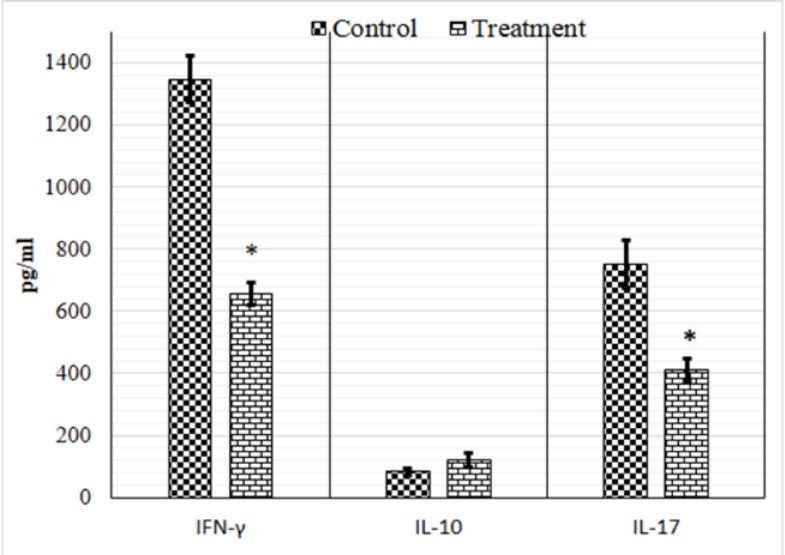
Cytokines production assay after treatment with aqueous extract of extract of tarragon. Spleen cells isolated from immunized mice with SRBC were cultured with 50µl of PHA (1 mg/ml) for 72h. The levels of IFN-γ, IL-17 and IL-10 in culture supernatants were determined after 72h by ELISA. The results were shown as mean ± S. D. (* P < 0.001, versus control mice

A significant decrease in secretion of IL-17 and IFN-γ by cells from tarragon extract-treated mice was observed compared to splenocytes from vehicle-treated group (Figure 1). The level of IL-10 diminished in the treatment group but this reduction was not significant (Figure 1). Therefore, the ratio of IFN/IL-10 and IL-17/IL10 were calculated. Interestingly, the proportions of INF-γ to Il-10 or IL-17 to IL-10 were significantly decreased. (Table 3).

**Table 3.** Cytokine levels ratio (IFN-γ/IL-10 and IL-17/IL-10

**Group**	**Cytokine ratio**	
	**IFN-γ/IL-10**	**IL-17/IL-10**
**Control**	24.52 ± 2.34	17.83± 2.1
**Treatment **	7.89 ± 0.9	6.7± 1.04
**p value**	< 0.001	< 0.001

Splenocytes separated from SRBC-immunized mice were incubated with 50µl of PHA (1 mg/ml) for 72 hr. The ratio of IFN-γ to IL-10 and the ratio of IL-17 to IL-10 in culture supernatants were determined after 72 hr. The data were shown as mean ± SD.

Phagocytosis, the production of reactive oxygen species (ROS) and nitric oxide are the main functions of macrophage to eliminate invaders and to maintain homeostasis. Results from this work showed that the phagocytosis of the zymosan particles was increased in the macrophages isolated from the peritoneum of the mice in the treatment group compared to macrophage isolates from the control group (Table 4). Nevertheless, macrophages isolated from treated mice significantly produced less nitric oxideand reactive oxygen species as compared to control group (Table 4).

**Table 4:** Effects of aqueous extract of *Artemisia dracunculus* on peritoneal macrophages activity

	**Nitric oxide** ^1^	**Phagocytosis ** ^2^	**NBT assay** ^2^
**Control**	66.1±5.4	1.12±0.11	1.33±0.07
**Treatment**	37.0±3.5	2.09±0.07	0.61±0.47
**P value **	< 0.001	< 0.01	< 0.01

Peritoneal macrophages isolated and nitric oxide production was determined using the Griess method, phagocytosis was assumed by natural red-stained zymosan and finally intracellular generation of reactive oxygen species (ROS) was measured by NBT reduction assy. 1 µM/ml, 2 Optical density

## Discussion

The main bioactive compounds of tarragon are essential oil such as estragol, cumarins, flavonoids, and phenolic acids. Pervious evidence showed that the major compound of Iranian essential oil and French tarragon contained estragol, whereas the essential oil of the Russian tarragon contained mainly methyl-eugenol (Obolskiy et al., 2011 ▶; Sharafati Chaleshtori al., 2013 ▶; Obistioiu D et al., 2014 ▶). Nevertheless, the European Union, Committee on Herbal Medicinal Products has advised a re-evaluation of herbal medications containing estragole and methyl-eugenol (Weinoehrl S, et al., 2012 ▶). The present study indicates that the aqueous extract of tarragon may be safer because it did not contain any detectable amounts of the two potentially harmful compounds. In this regards, the aqueous extract of tarragon was preferentially used in the following experiments.

Anti-inflammatory and anti-exudative activities of ethanol extracts of *A. dracunculus* have been determined in previous studies (Obolskiy, et al., 2011 ▶). Obviously, inflammatory response and immune function are completely intertwined. DTH is one of the typical response patterns of T cell-mediated immunity and causes T cell–dependent inflammation (Kobayashi et al., 2001 ▶). DTH is a major mechanism of defense against various intracellular pathogens and anti-tumor defense. Nevertheless, when the DTH reaction is inefficient in the removal of infection or is induced against harmless antigens, it can cause tissue injuries (Murdaca et al., 2011 ▶). In this work, we observed that treatment with the aqueous extract of tarragon significantly alleviated cellular immunity and concurrently potentiated humoral immunity following challenge with SRBCs. In general, cellular and humoral arms of immunity are reciprocally regulated. (Kobayashi et al., 2001 ▶). Therefore, increasing the humoral immune response following reducing DTH reaction in the treatment group is not far-fetched. 

DTH response is mediated by cooperation of Th17 and Th1 lymphocytes (Aranami and Yamamura, 2008 ▶; El-behi et al., 2010 ▶; Fletcher et al., 2010 ▶). IL-17 producing T lymphocytes (Th17) has a potent pro-inflammatory property (Korn et al., 2007 ▶) and plays a crucial role in the promotion and initiation of DTH induction (Kuerten and Lehmann, 2011 ▶). IFN-γ producing T lymphocytes (Th1) activate macrophages and determine tissue damage (Murdaca et al., 2011 ▶). Our data showed that the aqueous extract of tarragon significantly suppressed potent pro-inflammatory cytokines (IL-17 and IFN-γ) and consequently decreased the DTH reaction.

 IL-10, a cytokine with anti-inflammatory properties, plays an important role in limiting and terminating inflammatory reactions and subsequently preventing tissue destruction (Asadullah et al., 2003 ▶; Saraiva and O'Garra, 2010 ▶). IL-10 has long been believed to be produced only by Th2 cells, but further evidence revealed that IL-10 could be produced by a wide range of immune cells, including Th1, Th2 and Th17 cell subsets and Treg cells (Saraiva and O'Garra, 2010 ▶; Mannino et al., 2015 ▶). In other words, it is possible that CD4+ T cells produce both IFN-γ and IL-10 or both IL-17 and IL-10 (Trinchieri, 2007 ▶; Mannino et al., 2015 ▶). In this situation, the balance between the levels of pro-inflammatory and anti-inflammatory cytokines can determine the fate of immune responses (Mannino et al., 2015 ▶). Based on our data, the level of IL-10 decreased in the treatment group, but this reduction was not significant. However, the ratio of INF-γ to Il-10 or IL-17 to IL-10 was changed in favor of IL-10.

Monocytes/macrophages are the second most important players in DTH reaction (Kobayashi et al., 2001 ▶). Macrophages are remarkably diverse and plastic. Based on the environmental factors, they can undergo a reprogramming which leads to the emergence of a spectrum of distinct functional phenotypes (Kim and Hematti, 2009 ▶; Cho et al., 2014 ▶; Italiani and Boraschi, 2014 ▶). Classically-activated macrophages or M1 macrophages have potent anti-microbial and inflammatory properties and may contribute to the pathogenesis of inflammatory diseases. Alternatively-activated macrophages or M2 macrophages produce less pro-inflammatory mediators such as nitric oxide and reactive oxygen substances (ROS) and play a role in resolution of inflammation via trophic factor secretion and a higher phagocytosis ratio (Kim and Hematti, 2009 ▶; Italiani and Boraschi, 2014 ▶). Our results showed that macrophages isolated from treatment mice significantly produced less nitric oxide compared to control mice (Table 4). Reactive oxygen species (ROS) play an essential role in the elimination of invading microorganisms via phagocytes (Hamaliaka and Novikova, 2010 ▶). Nevertheless, when the production of ROS is excessive or inappropriate, ROS are involved in severe host tissue injury and participate in immunopathological conditions (Babior, 2000 ▶). In this study, treatment with tarragone significantly decreased the respiratory burst of macrophages challenged by opsonized yeast. Higher phagocytic activity is a property of anti-inflammatory M2 macrophages (Cho et al., 2014 ▶; Italiani and Boraschi, 2014 ▶). Our data similarly showed that the phagocytosis activity of macrophages was markedly increased in animals that received the aqueous extract of tarragon compared to control mice.

Immunomodulation is required when the host defense mechanism has to be activated in immunodeficiency conditions or when a selective immunosuppression is needed in autoimmune disturbances (Visavadiya et al., 2009 ▶; Mitra Mazumder et al., 2012 ▶). Immune deviation from cellular immunity to humoral responses and induction of anti-inflammatory phenotypes in effector cells such as macrophages have a substantial role in decreasing the extent of organ-specific autoimmune diseases (Aranami and Yamamura, 2008 ▶; Kuerten and Lehmann, 2011 ▶). The aqueous extract of tarragon which is free from potentially harmful estragole or methyl-eugenol, possesses immunomodulatory effects, *in vivo*. The immunomodulatory benefits of the aqueous extract of *A. dracunculus* may be partly due to the inhibition of pro-inflammatory cytokines (IL-17 and IFN-γ) and induction of anti-inflammatory macrophages. However, other mechanisms may also be involved, and these remain to be clarified. Collectively, these data suggest that the aqueous extract of tarragon may be used as a natural source to intervene the immune system.

## Note

*Please cite this paper as: *

Abtahi Froushani SM, Zarei L, Esmaeili Gouvarchin Ghaleh H, Mansori Motlagh B. Estragole and methyl-eugenol-free extract of *Artemisia dracunculus* possesses immunomodulatory effects. Avicenna J Phytomed, 2016; 6 (5): 526-534.
